# Strengthening close to community provision of maternal health services in fragile settings: an exploration of the changing roles of TBAs in Sierra Leone and Somaliland

**DOI:** 10.1186/s12913-017-2400-3

**Published:** 2017-07-05

**Authors:** Evelyn Orya, Sunday Adaji, Thidar Pyone, Haja Wurie, Nynke van den Broek, Sally Theobald

**Affiliations:** 1 National Primary Health Care Development Agency, Abuja, Nigeria; 2 Centre for Maternal and Newborn Health, Liverpool School of Tropical Medicine, Liverpool, UK; 3 College of Medicine and Allied Health Sciences Freetown, Freetown, Sierra Leone; 4 ReBUILD Consortium International Public Health, Liverpool School of Tropical Medicine, Liverpool, UK

**Keywords:** TBAs, Close to community providers, Maternal health, Sierra Leone, Somaliland

## Abstract

**Background:**

Efforts to take forward universal health coverage require innovative approaches in fragile settings, which experience particularly acute human resource shortages and poor health indicators. For maternal and newborn health, it is important to innovate with new partnerships and roles for Traditional Birth Attendants (TBAs) to promote maternal health. We explore perspectives on programmes in Somaliland and Sierra Leone which link TBAs to health centres as part of a pathway to maternal health care. Our study aims to understand the perceptions of communities, stakeholder and TBAs themselves who have been trained in new roles to generate insights on strategies to engage with TBAs and to promote skilled birth attendance in fragile affected settings.

**Methods:**

A qualitative study was carried out in two chiefdoms in Bombali district in Sierra Leone and the Maroodi Jeex region of Somaliland. Purposively sampled participants consisted of key players from the Ministries of Health, programme implementers, trained TBAs and women who benefitted from the services of trained TBAs. Data was collected through key informants and in-depth interviews and focus group discussions. Data was transcribed, translated and analyzed using the framework approach. For the purposes of this paper, a comparative analysis was undertaken reviewing similarities and differences across the two different contexts.

**Results:**

Analysis of multiple viewpoints reveal that with appropriate training and support it is possible to change TBAs practices so they support pregnant women in new ways (support and referral rather than delivery). Participants perceived that trained TBAs can utilize their embedded and trusted community relationships to interact effectively with their communities, help overcome barriers to acceptability, utilization and contribute to effective demand for maternal and newborn services and ultimately enhance utilization of skilled birth attendants. Trained TBAs appreciated cordial relationship at the health centres and feeling as part of the health system. Key challenges that emerged included the distance women needed to travel to reach health centers, appropriate remuneration of trained TBAs and strategies to sustain their work.

**Conclusion:**

Our findings highlight the possible gains of the new roles and approaches for trained TBAs through further integrating them into the formal health system. Their potential is arguably critically important in promoting universal health coverage in fragile and conflict affected states (FCAS) where human resources are additionally constrained and maternal and newborn health care needs particularly acute.

**Electronic supplementary material:**

The online version of this article (doi:10.1186/s12913-017-2400-3) contains supplementary material, which is available to authorized users.

## Background

In the past two years, there has been a growing commitment to the goal of universal health coverage (UHC) and wide reaching and high-level discussion about the centrality of UHC to the post-2015 Millennium Development Goal agenda and the development and formation of the Sustainable Development Goals. Taking forward UHC will be futile without specific effort and action in fragile states. While definitions and figures vary, some 1.2 billion people are estimated to live in fragile and post-conflict settings [1]. It estimated that ‘one third of the global poor lived in fragile states in 2010, and projections indicate that roughly half will do so by the year 2015’ [2]. In fragile settings, access to equitable and quality health services is not only vital, but of huge importance for rebuilding the social fabric of countries [1].

The health workforce is a key health systems building block that underpins the expansion of health services and UHC efforts. Most countries in the global South have a shortage of formal health workers and are increasingly looking to a range of close to community providers (e.g. community health workers (CHWs), village midwives and traditional birth attendants (TBAs)) to fill the gap and reach the poorest and most marginalised individuals, households and communities. Close to community providers are arguably critical players in fragile settings, settings where human resource shortages are particularly acute as health workers may have been killed during conflict, fled the country or simply unable to reach their work places as they are located in hard to reach areas. Maternal deaths and under-five mortality rates are much higher in fragile and post-conflict states and this is an urgent area for action [3–6]. Despite their potential, there is limited literature assessing the different actual and potential role of close to community providers (such as TBAs) in promoting maternal health in fragile settings.

The role of TBAs in maternal and newborn health has been widely debated. A TBA is defined by WHO “as a person who assists the mother at childbirth and who initially acquired her skills delivering babies by herself or by working with other birth attendants” [7]. TBAs have been in existence since at least the nineteenth century and were additionally trained to conduct safe deliveries in order to address the shortage of skilled birth attendants especially in developing countries where maternal mortality remained high [8, 9]. The evidence shows that TBAs have made no significant change in the reduction of maternal deaths [10–12], although some reductions in perinatal mortality attributable to TBA training has been documented [13]. Hence, as far back as 2002, the World Health Organization (WHO) suggested a rethink of the roles and responsibilities of TBAs within their communities and suggested they could work as health promoters to improve the utilization of skilled birth attendants, rather than directly as primary care providers who deliver babies themselves [14]. This has led to several new initiatives that partner with TBAs to improve maternal and newborn health [15, 16].

Sierra Leone and Somaliland (a self-governing autonomous region of Somalia) are both fragile settings with poor maternal and child health indicators (Table 1); there is no sub-national data available in either setting on these health indicators. In 2011, there were relatively better indicators in Sierra Leone than in Somaliland and this is in part due to the free health scheme for mothers and under-fives [17]. However, in both settings TBAs are still commonly used due to the trust they enjoy at community level and the shortage of Skilled Birth Attendants (SBA). Both contexts are not only resource-constrained but have in the recent past experienced widespread social conflict, which has led to shortages of skilled birth attendants and a consequent increasing reliance on TBAs, massive displacement of women and their families and exacerbated household poverty. In the case of Sierra Leone, recent gains in health systems strengthening and maternal and child health indicators have been undermined by Ebola, which like conflict, brings wide reaching challenges to all levels of the health system.

**Table 1:** Maternal and newborn data for Somaliland and Sierra Leone

Indicator	Somaliland [42]	Sierra Leone [43]
MMR/100000 live births	1000 to 1400^a^	857
Neonatal mortality rate/1000 live births	42	39
Antenatal care coverage (4 or more)	14.8%^b^	76%
Institutional deliveries	30.6%^b^	54%
Skilled birth attendant utilization	44. 1%^b^	60%

^a^Source: Somaliland MoH (2011)

^b^Source: MICS 2011 (UNICEF et al., 2014)

Health Poverty Action (HPA) is a non-governmental organization (NGO) which aims to strengthen “poor and marginalised people in their struggle for health, prioritising the communities almost everyone else in the world has forgotten” [18]. HPA works in 12 countries in Africa and Asia. Within Somaliland and Sierra Leone, HPA prioritized a focus on maternal health as this is a key priority area in both contexts. HPA has worked with partners to design and implement programmes that work in partnership with TBAs in ways that build on their trusting relationships with communities to try to improve maternal health experiences and indicators. In this new model, TBAs do not actually deliver babies, but rather give information and support and encourage women to give birth in health centres. The details of both programmes are as follows:

### Somaliland

The TBA programme is part of a larger project named “Improving the Reproductive and Sexual Health of Internally Displaced People, Maroodi Jeex, Somaliland”. It was implemented by Health Poverty Action (HPA) in partnership with the Liverpool School of Tropical Medicine (LSTM) and the Somaliland Ministry of Health (MOH) from 2008 to 2012. The project composed of supporting increased SBAs and creating an enabling environment through new partnerships with TBAs. The programme of work included improving all five maternal and child health (MCH) centres and one referral hospital in Hargeisa with infrastructure rehabilitation, supply of medical equipment, drugs and consumables, running costs and salary top-ups for staff working in the maternity area and competency-based training for SBA in skilled birth attendance and emergency obstetric and newborn care. TBAs were trained as “health promoters” and “birth companions” and provided specific links to MCH centres. The trainings focused on their understanding of the dangers of home births, the benefits of facility delivery and the need for prompt referral of all pregnant women to the MCH centres. Additionally, TBAs received USD$5 for each patient referred or escorted to any of the five designated MCH centres.

The introductory trainings of TBAs lasted for 3 days with an emphasis on the need for antenatal care, understanding the dangers of a home birth, the benefits of facility delivery and a professionally trained SBA, the need for prompt referral of all pregnant women to a maternal and child healthcare facility for care, the importance of companionship and how to help women who were afraid of a facility-based birth or of complications. In addition, the TBAs visited the healthcare facility and were oriented in the services provided at facility level as well as introduced to the staff working there as SBA. Refresher training was provided one year after the initial training.

### Sierra Leone

Like Somaliland, the programme involving TBAs was part of a three-year programme (2012–2015) entitled “Building capacity for the improvement of infant and maternal health in northern Bombali, Sierra Leone” [19]. The programme was also implemented by Health Poverty Action (HPA) in partnership with Liverpool School of Tropical Medicine (LSTM) and the Sierra Leone Ministry of Health (MOH). The programme aimed to strengthen the health system by training health staff in emergency obstetric and newborn care, improving the Maternal and Child Health (MCH) centres and a referral hospital in Kamakwie by restructuring, supplying medical equipment, drugs and other consumables. TBAs were trained to become “Maternal Health Promoters” in all the 28 Public Health Units (PHUs) in the five (5) chiefdoms. The training of the TBAs was similar to that in Somaliland, as the same guidelines (adapted from the Maternal and Child Health training programme 2000) in Sierra Leone were utilized.

In Sierra Leone, nine (9) TBAs were selected for each of the 28 PHUs and the training lasted for five days in each PHU and pictorial training manuals were used to train the TBAs as majority of them have no formal education. They were trained on 23 different topics ranging from danger signs in pregnancy to family planning methods. Criteria that were used to select TBAs were women aged between 30 and 55 years and those willing to work for the community. This training focused on change of role from taking deliveries to a non-delivery role, the gains of facility delivery and strategies to promptly refer all pregnant women to the PHUs. Trained TBAs received USD$3/month and bags of grain (also monthly) as remuneration. This new approach was rolled out at the same time as village chiefs enforced by-laws stating that any pregnant woman found delivering at home (along with the assisting maternal health provider (MHP) as appropriate) will be made to pay a fine of 50,000 Leones (USD$10). Herschderfer et al. mentioned in a research conducted in Sierra Leone that it is unclear how these by-laws came into existence although by 2012 they appeared to have been established in most parts of the country [19].

Our study aims to explore the new role of TBAs promoted by HPA within 2 different African contexts to understand the strengths and weaknesses of the approach and generate lessons that draw from more than one context. Specifically, we aimed to understand the perceptions of trained TBAs in their new roles and the perspectives of communities and other stakeholders involved in delivery of maternity care and generate important insights about strategies to engage with TBAs to promote maternal and newborn health in fragile and conflict affected state (FCAS) settings.

## Methods

### Study design

A qualitative study design was deployed to generate knowledge from discoveries made from individuals’ perceptions and experiences [20]. In both settings, face to face in-depth interviews (IDIs) and focus group discussions (FGDs) were used with purposively sampled participants to understand views, experiences and perspectives on TBAs as health promoters and birth companions in the study settings. IDIs enabled the researchers to probe in-depth with respect to a particular individuals’ experience whereas FGDs allowed for a better understanding of the role of group dynamics in shaping individual experiences and decisions with respect to child care delivery [21]. The study design combined primary qualitative research with desk based review of project documents and training materials. The studies were carried out separately, in Somaliland the qualitative research took place in April 2013 [22] and in Sierra Leone, June 2014, just prior to the Ebola outbreak. The qualitative study designs were similar, and we have merged the two analytical frameworks to enable a comparative analysis and generate wider insights on the changing role of TBAs in two different fragile settings.

### Study settings

In Sierra Leone, the study was carried out in two out of the five chiefdoms in Bombali district namely Sella Limba and Tambakha. The Primary Health Units (PHUs) selected were Kamawonie Maternal and Child Health post (MCHP) in Sella Limba and Fintonia Community Health Post (CHP) Tambakha chiefdoms respectively. These chiefdoms were selected because one was hard to reach and the other easily accessible. These two chiefdoms speak different languages: Limba in Sella Limba and Susu in Tambakha. In Somaliland, the study involved health facilities (Sheikh-Noor, Mohammed Moge, Saxaardid, Iftin and Abdi Eden Health centers) in Maroodi Jeex region of Somaliland. These health facilities had as catchment areas internally displaced persons (IDP) and returnee communities camped closed to the facilities. Project documents, baseline and interim reports from 2009 to 2012 and training reports were reviewed to better understand the background, objective and progress of project. In Somaliland data collection took place towards the end of the project; whereas in Sierra Leone it took place near the beginning of the project, as this was done about a year after the training of the MHPs.

### Sampling

The sampling approach in both contexts was purposive aiming to capture a range of views and perspectives by age, experiences and location to enable “symbolic representation” [23] of the broader constituencies. For example, in both contexts, both younger and older TBAs with different levels of experience were included to understand their different experiences. In both countries, the study participants consisted of key players from the Ministries of Health, programme implementers (HPA), trained TBAs and women who benefitted from the services of trained TBAs. In Somaliland, interviewees from the Ministry of Health (MOH) in Somaliland included central level Ministry staff, staff from the Regional Health Office (RHO), and healthcare provider (SBA) from each of the five MCH centres, and in Sierra Leone, interviewees included staff from the MOH, Regional Health and district offices. Providers of health care from the two PHUs were also interviewed.

In Sierra Leone, the Chief of each of the chiefdoms was also interviewed while in Somaliland, a group of women who did not benefit from the trained TBAs also participated in the study. Table 2 provides details of the study participants in each country and the data collection method utilized.

**Table 2:** Study participants and data collection methods

Participants	Somaliland	Sierra Leone	Data collection method used
Users of service	Recently delivered women: Abi Eden: 6 Iftin: 8 Mohammed Moge: 8 Saxaardid: 6 Sheik Noor: 6	Pregnant/recently delivered women: Kamawonie: 7 Fintonia: 8	Focus group discussion: 10 in Somaliland 2 in Sierra Leone
TBAs trained as maternal health providers (referred to as trained TBAs)	Abi Eden: 6 Iftin: 8 Mohammed Moge: 6 Saxaardid: 6 Sheik Noor: 6	Kamawonie PHU: 4 Fintonia PHU: 3	Focus group discussion (Somaliland) In-depth interview (Sierra Leone)
Health workers in health care facilities	1 health worker from each of the 5 health facilities	1 each from Kamawonie and Fintonia	Key informant interview (Somaliland) In-depth interview (Sierra Leone)
Health care managers	1 MoH staff & 1 Staff of the regional health office	2 MoH staff	Key informant interview (Somaliland) In-depth interview (Sierra Leone)
HPA Staff	1	2	Key informant interview (Somaliland) In-depth interview (Sierra Leone)
Village chiefs	Nil	1 each from Kamawonie and Fintonia	Key informant interview
Total participants	74	30	-

Checklists that guided the in-depth interviews and focus group discussions

### Data collection process and analysis

The qualitative project in Sierra Leone was led by EO; and in Somaliland by TP and SA. In both contexts stakeholder interviews were conducted in English while most FGDs and some in-depth interviews were carried out in local languages depending on the participants’ preference (Somali for Somaliland, Limba and Susu for Sierra Leone) in offices and health centres. The checklists that guided these interviews and topic guides were pre-tested are included can be found in Additional files 1, 2 & 3. The interviews involved only participants, researchers and translators/research assistants. Researchers worked with translators/research assistants who had been appropriately trained in qualitative research approaches. In Sierra Leone, the training of the interviewers lasted for four days, the interviewers were given a brief on the study, and then supported in the use of non-leading and open questions and the importance of paying attention to body language. They were then asked to sign a confidentiality agreement. A detailed explanation of the training process can be found in (Additional file 4).

In Somaliland, KIIs were conducted by SA and TP while FGDs were conducted by the trained moderators. TP and SA facilitated a half day orientation workshop on how to conduct FGDs for moderators, note takers and translators at HPA office in Hargeisa. One national research officer from the MOH also facilitated the training particularly for explanation in vernacular language. Moderators included staff from the MOH, Ministry of Planning and HPA project staff. During the workshop, basic concepts underpinning FGDs were discussed. The moderators and note takers were given tape recorders and gained familiarity in using them. Orientation on the FGD topic guides translated into Somali was facilitated by the research officer from the MOH. Comments on discrepancies in translation between the Somali and English version guides were resolved during the orientation workshop.

Both FGDs and interviews were audio recorded and field notes made during the interviews following informed consents from the study participants. FGDs were transcribed verbatim after verbal translation by the interpreter in country. The quality of translations was checked by research assistants before transcribing them into English. Recording of key informant interviews were also transcribed verbatim. Researchers listened and checked all the recordings against the transcripts before analysing the data. Data from both contexts were analysed following the thematic framework approach: synthesizing and charting the data against the key themes that emerged [23]. Data were analysed independently for both contexts. NVIVO 10 was used in the analysis of the Sierra Leonean study, and example of the analysis process here can be found in Additional file 5. In the case of Somaliland, we have used Microsoft Office and Excel to develop thematic framework and MindGenius Business Version 6 was used to develop maps of themes and sub-themes. Themes were derived from the data and E0 and ST coded the data in Sierra Leone and Additional file 6 shows the coding tree at the end of the article while in Somaliland TP and SA coded the data. For this paper a comparative analysis was undertaken reviewing similarities and differences across the two different contexts through a process of joint meetings and discussion.

### Ethical consideration

The qualitative study in Sierra Leone received ethical approval from the Sierra Leone Ethics Committees; and for Somaliland from the Somaliland Health Research Ethical Clearance Board. Both studies received ethical approval from the Liverpool School of Tropical Medicine Ethics Committee (Additional file 7). Informed consent was obtained in all cases and the utmost care was taken to ensure confidentiality throughout the research process. The informed consent taken in both contexts was written (although where participants were illiterate, the facilitators explained the information and process verbally and asked them to put their thumb prints on the consent forms).

## Results

We present the four key themes which emerged from the joint analysis of participants’ perceptions and experiences of trained TBAs’ new role in promoting maternal and newborn health following the intervention supported by HPA in rural contexts in Sierra Leone and Somaliland, and these have been used to structure the results section Theme 1 presents participants’ perspectives on the value of the training received; theme 2 the impact of the trained TBAs new role; theme 3 opportunities to strengthen integration of trained TBAs into the health system and theme 4 the challenges to realizing and sustaining trained TBAs new role. In the results SL is used for shorthand for Sierra Leone and SMLD for Somaliland.

Theme 1: Perspectives on the value of the training received

Providing training to TBAs was considered an important step by all participants. An IDI with a Somaliland MOH staff acknowledged the shortcoming of TBAs in their usual role as providers of maternity care. These two quotes refer to TBAs before they were trained to become MHPs.

*“They do provide some advice and do referring if they (pregnant women) are anaemic to the nearest health post, conduct deliveries when the time of delivery is due, if the mother [is] not complicated and they feel any complications they refer to the health centres. That was their primary role but overall it was so that their role had not much helped.” (IDI, MOH representative, SMLD)*

During an FGD session, women voiced their reservation about the knowledge and skills of the TBAs.

*“[A] TBA doesn't know more than us, she just comes and catches the baby, she only prays to Allah, she doesn't know the danger signs. Mostly they try to do home delivery until it's very late.” (FGD, SMLD)*

All trainees interviewed valued their training; which was seen as important in upgrading skills, level and in some cases as a boost to means of livelihood. For example:

*“To increase my educational qualification and to give me what I can buy soap with.” (IDI, F, trained TBA, SL)*

Some Trained TBAs felt that the process of being engaged in training meant that they had earned more respect from the women and their husbands and were more recognized at the community level and beyond their immediate village.

*“This job has made me very popular and I now have a lot of respect in the village, even the people who don’t want to respect me do when I talk to them. Also, the training has exposed me. .. it has made me meet with different people, pregnant women and their husbands and they call me in Kamakwie and Makeni for meeting.” (IDI, F, trained TBA, SL)*

A Somaliland TBA reflected on what she has gained from the training which in her view has changed the way she worked significantly.

*“Before this project, we were delivering the mothers at home. We were not recognizing the danger signs … we met a lot of complicated cases, even when some mothers were dying at home because we were just waiting for the baby. We would not know…. Just we were thinking that everyone is having a normal delivery. But, after this project and during this we got a lot of trainings, we know danger signs.” (FGD, TBA, SMLD).*

Theme 2: Perceptions and experiences of the impact of the training and the new TBA role.

In both countries, the training of TBAs was perceived to have had a positive impact on the communities by increased community sensitization on the need for utilization of health care services, by women and other members of the community and reduction of maternal and child deaths.

In Sierra Leone, pregnant women participating in focus group discussions discussed the benefits of utilizing hospital services and discontinuing deliveries at home. Many of them spoke about the advice trained TBAs had given them about the dangers of delivering at home and the importance of health center utilization.

*“Before the training, pregnant women were dying but since this project introduced the MHPS (trained TBAs), they encourage us to go to the clinic. They advise us to come to the hospital because of well body and that if we stay at home maybe we might have sickness in our bodies and we won’t know.” (FGD, Pregnant woman, SL)*

Some of the trained TBAs also said that the pregnant women were unaware of the consequences of giving birth at home but now they know because of their training.

*“At first plenty pregnant women died because there is no sensitization and plenty go astray, some of them died when there are giving birth and some lost their babies but now it is different.” (IDI, F, trained TBA, SL)*

Most pregnant women in Sierra Leone had very positive views about the roles of the trained TBAs and a few even pointed out some deficiencies of TBAs who have not been trained. Most of the women spoke of the roles of the TBAs assigned to their villages and many said the trained TBAs go to their houses regularly to check how they and their babies are doing and ensure they take their medicines.

*“The MHP (trained TBA) goes round our houses to check if we are taking the drugs given to us, if you are not taking it she will tell you they are tablets that will help you deliver well so you have to take them. She does this always.” (FGD, Pregnant woman, SL)*

In Somaliland, participants also provided similar perspectives. In their view, the training of TBAs has also influenced the health seeking behavior of pregnant women in their community.

*“Now, most pregnant women understand to come to the MCH centre and have ANC. There are women who go directly to health facilities. Numbers of home deliveries are reducing in the last 5 years… TBAs can now identify risk cases, can do basic reporting orally… or she asks her child to write.” (IDI, Health worker, SMLD)*

Within Sierra Leone, and as part of the post conflict health reconstruction process the Free Health Care initiative was launched in 2010 with the promise of free health care for pregnant and lactating women and under-fives. Analysis of interviews with health workers and Ministry of Health staff in Sierra Leone highlighted that even with the free health care initiative there were still many pregnant women delivering at home but since the training was completed there has been an increase in the utilization of services in the PHUs.

*“I say it has changed because before we had a lot of home deliveries more than institutional deliveries, but now we have a form that is filled by the health facility staff…. you know. It contains deliveries conducted, assisted by Trained TBAs and at the end of the month they collate all the data and send them to this office. From these we can see that deliveries conducted in health facilities (are) higher than deliveries conducted in non -health facilities.” (IDI, M, MOH staff, SL)*

Most pregnant and newly delivered mothers in Sierra Leone emphasized that trained TBAs had stopped taking deliveries at home and that pregnant woman were utilizing the health centers.

*“No more delivery at home, if the MHP (trained TBA) knows you are pregnant she tells you whenever you are ready to deliver to just knock on her door even if it’s at midnight she will take you to the center.” (FGD, F, Pregnant woman, SL)*

Different participants discussed similar positive increases in health facility delivery post training Somaliland.

*“Before the project, we used to deliver pregnant women at home. Some of them have bleeding and other complications, like convulsions and blood pressure, bleeding and might die at home, but now everything has changed.” (FGD, TBA, SMLD)*

*“Most deliveries occur in health centres…. TBAs know that home delivery is risky.” (IDI, Health worker, SMLD).*

One key informant from Somaliland gave an account of a referral for utilization she witnessed.

*“I saw one mother's referral [...] to the MCH and I was at the MCH at that time. She [trained TBA] took one mother, with three babies in her abdomen, and she delivered safely in MCH and if that TBA had not transferred to us maybe the mother [she] would have had some [risk] problem....and the mother and her family, they were very happy.” (IDI, Health worker, SMLD)*

With regard to maternal and child deaths, almost every participant in Sierra Leone mentioned that there had been a noticeable reduction in the deaths of pregnant women and their babies compared to what they were experiencing prior to the training of the Trained TBAs.

*“It is not easy now to hear that a pregnant woman died and even the babies are no more dying.” (FGD, Lactating mother, SL)*

The health center staff said since the training there has been no death recorded at all in the health center.

*“The number of pregnant women dying has reduced, in fact since this training no pregnant woman has died here.” (IDI, M, Health worker, SL)*

The findings from Somaliland are complemented by the output indicators (proportion of TBAs’ referral and facility delivery rates) from the project documents. During the first six months of the training, 56% of total deliveries were referred, while 72% were referred in second year and 67% in the third year of the project. On the other hand, the desk review of project documents highlighted that total numbers of women received maternity care at the five maternity centers increased from 779 in 2009 to 3296 in 2012. Routine project data elicited that there has been a steady rise in facility-based deliveries from baseline (2009) peaking by mid-2012.The SBAs working at those maternity centers reported that the changing role of TBAs and linking up with the TBA with the facility contributed partly to the increase in skilled deliveries at health facilities.

In Sierra Leone, the research was carried out within the first year of the training hence, and the number of referrals made by MHPs from June 2013–April 2014 which was 28,640 (HPA and Ministry of Health).

The analysis in both contexts highlight how all different participant groups from pregnant women, to trained TBAs to key informants perceive the training has had a positive impact on community sensitization, health center utilization and maternal and child health. Within the Sierra Leonean context, the training and new roles for TBAs coincided with new bylaws that were being enforced by local village chiefs: any woman found delivering outside the health center, and those supporting her (e.g. TBAs trained or untrained) would be made to pay a fine of 50,000 Leones, (approximately USD$10). It was unclear how often this was enforced but it emerged as an issue within the FGDs and is likely to have also had an impact on increased numbers of health center deliveries.

Theme 3: Opportunities to strengthen integration of trained TBAs into the health system.

Within the Sierra Leonean contexts, trained TBAs and key stakeholders mentioned that there were now in an era of a good working relationship between trained TBAs and the health center staff. Trained TBAs felt that there were increasingly cordial relationships between them and health workers. One trained TBA explained that whenever she brings women to the health center, she feels happy because they are given prompt care.

*“We (Trained TBAs and health staff) are working well together in unity for the community and when someone wants to give birth or I come with a baby here they attend to them immediately and give them good attention. I feel very good about that.” (IDI, F, Trained TBA, SL)*

This improved relationship has also been echoed in Somaliland. TBAs developed relationships with skilled birth attendants, and could call and bring women along to health centres without fear of intimidation. In Sierra Leone, SBAs were sensitized to the new role and some of them were involved in the training of the TBAs to become MHPs. Some TBAs were invited to help in the facilities. Furthermore, TBAs received feedback from SBAs on some of the women they had referred or escorted. One TBA expressed her thoughts on this.

*“When accompanying the woman to the health facility, the staff receives us and welcome us very well.” (FGD, TBA, SMLD)*

Health workers are beginning to increasingly recognize the role of TBAs in their communities and the value in working with them to reach the community. As one health worker in Somaliland surmised;

*“No-one can reach to a community if there's no TBA.” (IDI, Health worker, SMLD)*

Theme 4: Challenges to realizing and sustaining trained TBAs new role.

### Distance and problems with transportation

Participants from both contexts highlighted the challenge of distance and transportation. In Sierra Leone, most trained TBAs interviewed lived more than 2 miles away from the PHU and either had to walk with heavily pregnant women or find motorbikes if resources were available. In Somaliland, most pregnant women and newly delivered mothers said means of transportation to the health facility was a big challenge. The problem of transportation was not tied to insecurity but related to hard to reach areas in the region especially in Tambakha in Sierra Leone where there are a lot of rivers.

*“The biggest obstacle is transport, we know MCH centers are free but you need transport to take (you from) your home, bring you to the health facility…. After that you need someone to bring your lunch, that person also needs transport. Again, I have four young children; three of them are very small. There will be no one if my husband comes to the facility with me.” (FGD, Woman, SMLD)*

In both contexts, transport challenges were compounded by the distances to heath facilities. Within Sierra Leone, health staff in one of the chiefdoms pointed out that about 42 villages accessed the health facility and some of these villages are about 25 miles away from the facility.

*“One problem is distance and also Tambakha is in the red line in terms of development and index in this country. People stay far away so encouraging them to come to the health facility is a problem because some do walk long distance of 20 miles, 25miles with very rugged roads.” (IDI, M, Health worker, SL)*

#### In Somaliland

*“Our health center is quite far for some women which is about 10-20 kilometers.” (IDI, Health worker, SMLD)*

Following discussions with MOH and HPA staff, we found that the involvement of the MOH from the planning of the training to its execution was seen as an opportunity to engage MOH as they aim to continue to support the Trained TBAs after the programme ends.

*“We are the Ministry of Health and we are here forever and NGOs operate with funds so when the project finishes, they forget about that project but the fact that they involved the Ministry of Health and we are doing everything together. Even when they finish we will step in to make sure we continue to support the health promoters.” (IDI, M, MOH Staff)*

### Remuneration

The level of incentives given to trained TBAs by HPA (USD$3 per month in SL/USD$5 per month in SMLD) was perceived as insufficient in both contexts. In Sierra Leone, this emerged as a key issue from every participant that was included in the study except the chiefs. For example:

*“Really compared to the amount of work the money is small because their number was large and it was out of the money that we removed to give them the group incentive so the amount is reduced. So, they do get only 15,000 Leones every month.” (IDI, F, HPA Staff, SL)*

#### In Somaliland

“It was only USD$5 and you know that USD$5 wouldn’t do anything for you.” (FGD, TBA, SMLD).

*“At least it (the incentive) helped…. The TBAs will say that the incentive that is given to them is not matching the standard they expected or the living conditions but still without the incentive, it (the project) would still have been successful as it is today. (Yes) but that needs to be reviewed, we even recommended it during that time. It depends on various conditions and the global recession, economic recession, it may not make that feasible but still it is one aspect of motivation or encouraging the role and active participation of the community and health promoters as well.” (IDI, MOH representative, SMLD)*

In both contexts health workers highlighted how being trained and receiving small incentives may actually have reduced the overall livelihoods of TBAs as compared to the time when they delivered at home and received direct payments from families following a successful delivery.

*“Imagine that when women were delivering in their homes in a day they get like 20,000 Leones so this 15,000 is too small for them but it will be difficult to sustain the project if we decide to give them a huge amount.” (IDI, M, HPA Staff, SL)*

*“Before, the TBA, when she delivered at home she would take some amount of money from the mother……. And HPA tried to change that habit and the HPA gave small benefits for TBAs…. TBAs were given USD$5 for each mother.” (IDI, Health worker, SMLD)*

### Disruption of farm activities and opportunity costs of being a trained TBA

Within Sierra Leone most trained TBAs interviewed complained of disruption of their farm activities since they received the training, as they always must leave their work to accompany pregnant women and their babies to the health center. Those who did not voice complaints about disruption in farm activities were the older ones who preferred to stay at the health centers with the health staff and had probably stopped working on their farms.

*“Since after the training I only go to farm sometimes and most times the women call me when I am on the farm so I have to leave my work and follow them to the center.” (IDI, F, MHP, SL)*

Within the focus group discussion pregnant/newly delivered mothers confirmed this saying:

*“The trained TBAs even leave their farm work to come with us. If someone complains of any problem, they leave their work and take that person to the center.” (FGD, F, Pregnant woman, SL)*

Within the Somaliland context disruption of farm activities did not emerge as a concern, trained TBAs were mostly internally displaced people and who did not have access to land and engage in farming. These are cattle rearing people and in this context, this is largely a male role.

## Discussion

Our qualitative analysis in two different fragile country contexts both confirmed that with appropriate training and support it is possible to change the behavior and practices of TBAs so that they become part of a partnership with formal health providers and have stronger links with health facilities and health systems. To our knowledge this is the first paper that has explored perspectives on new approaches to TBAs roles in two different contexts. Trained TBAs can interact effectively with their communities, and in two different contexts were perceived to help overcome barriers to acceptability, utilization and contribute to effective demand for maternal and newborn services and impact on utilization of skilled birth attendance. Trained TBAs were able to perform non-clinical roles, linking women to care, and this has the potential to transform health seeking behavior and health outcomes for mothers and newborns in fragile and conflict affected settings and beyond.

Training and reorientation was essential for TBAs to adopt their new roles. The training provided in Somaliland and Sierra Leone focused on early referral of women for delivery at a health facility and providing companionship during labour and delivery. This contrasts with previous training modules, which emphasized building TBAs’ skills to safely conduct deliveries, recognize and refer women with complications [24]. In the two different contexts, and at different stages in the implementation of the projects (later in the HPA project cycle in Somaliland and earlier in Sierra Leone) the training of TBAs was viewed positively by the trained TBAs themselves, the health workers and pregnant and newly delivered women at community level.

Improved utilization of SBAs and reduction of home delivery are key maternal behaviours that could arguably improve outcomes for mothers and their newborns [25, 26]. The TBA training and reorientation to perform new roles - including referral and companionship – were perceived by different stakeholders (including women, TBAs, health providers and participants from the Ministry of Health) to have impacted maternal behaviours. This is consistent with the findings of a recent systematic review which showed that women were more likely to be referred if they lived near a TBA trained to refer compared to women who lived elsewhere [12]. Byrne and Morgan’s systematic review demonstrated that when TBAs are trained and supervised, they are more likely to refer complicated cases [16]. With the approach taken in both Somaliland and Sierra Leone, TBAs were trained and required to refer all women (with or without complications) for a facility-based birth.

Qualitative perspectives from trained TBAs, communities and key stakeholders from Ministries of Health and health centres in both study countries confirmed that trained TBAs had a positive impact on community sensitization for uptake of health services by mothers. Most trained TBAs mentioned during interviews their ‘commitment’ to the community in which they lived and worked. TBAs, like other close to community providers, are embedded in communities, and often have trusting and respected relationships with women at the community level and shared language and cultural framing of health issues [21]. In addition to being accompanied by TBAs, it is also possible that pregnant women were motivated to use the health care facilities because of the importance they attach to traditional norms and values, of which listening to the advice of trained TBAs whom they have a lot of respect for and feel comfortable with, is a component. A study that assessed the contributions of TBAs in maternal health in Ghana confirmed that TBAs enjoyed a lot of trust and respect in their communities and were in a position to influence women’s health behaviour [8]. Hence training and partnerships with TBAs which builds on their embedded and trusted community positionality has great potential.

The recent outbreak of Ebola in Sierra Leone changed the landscape on multiple fronts and brought additional human resource constraints. At the peak of the outbreak, health workers were severely affected, and many have lost their lives and those who remained faced multiple challenges in responding to the epidemic, due to limited prior knowledge on infection prevention and control practices. Sierra Leone’s fragile health system has been further weakened and it is likely that recent gains made in maternal health are being eroded. The level of mistrust between health service users and health professionals has increased as the virus continued to spread [27]. In the post Ebola reconstruction phase, efforts must be placed on bridging this gap. At the peak of the outbreak, it was documented that women and children are staying away from hospitals and health centres due to the fear and stigma associated with Ebola [28]. The additional burden posed on the already fragile health care delivery infrastructure by the Ebola virus disease epidemic and the underutilization of health facilities by this group puts them at even greater risk for adverse outcomes. There are reports of pregnant women dying at home of preventable illness; an estimated 400 mothers died between 20th May 2014 and 15th July 2014 from preventable illness [29], with an estimated 31% decline in institutional deliveries from May to Sept 2014 resulting in a corresponding rise in maternal case fatality rate from 1.27 to 3.08 in facilities providing comprehensive emergency obstetric and newborn care, which peaked at 3.48 at the height of the outbreak in Nov 2014 [30]. The impact of Ebola on trained TBAs is largely unknown, but they are very likely to have been affected as their gendered caring roles within households and communities means they are especially vulnerable to infection. In Liberia, it is estimated that 75% of Ebola cases are female and in Sierra Leone women have comprised 55 to 60% of the dead [31]. Lessons learnt from containing the Ebola outbreak in Uganda in 2001 highlighted the importance of rebuilding trust and collaborative working relationships with different community groups and structures and the importance of community health workers in this respect [32]. Close to community providers, such as TBAs, embedded within and trusted by communities are arguably strategically placed to help rebuild trust, in the post Ebola reconstruction phase. Strategies to support and sustain them will be particularly critical.

So how can trained TBAs be best supported to realize their roles? Our analysis shows that during the fieldwork period a cordial working relationship between health centre staff and trained TBAs seemed to have evolved in both Sierra Leone and Somaliland. As stated earlier, most of the SBAs were involved in the training of the TBAs and they were trained on topics that SBAs already practiced. Most TBAs reported that they were well-received and well-treated by the healthcare workers (skilled birth attendants) each time they referred or escorted a woman and this was highly valued. These positive reports contradict the report of abusive relationships between health care workers and TBAs that have been previously documented [33]. Findings from a systematic review have also emphasized the need for a cordial relationship between Trained TBAs/TBAs and health workers [16].

In resource-constrained, largely donor dependent settings and especially fragile settings like Sierra Leone and Somaliland, remuneration for TBAs, and other cadres remains a contentious issue. This is partly attributable to the fragility of the economy and limited resources available for health care delivery. The gains and pitfalls of paying TBAs for their services has been persistently debated [34] and emerged as a key theme in both contexts in our study. Traditionally, TBAs have had no formal payment package for their services, rather depending on the goodwill accorded to their roles and payment in cash or kind by their clients [35, 36]. In our study, the incentives paid to TBAs as they performed their new roles in both countries were largely viewed to be inadequate; and in both contexts TBAs may receive less overall remuneration in their new roles (i.e. USD$3/month in Sierra Leone and USD$5/month in Somaliland may well be less than what they would have received in donations/in kind delivering babies at home in the past). In Sierra Leone participants (women from the community and trained TBAs) felt that the new role given to the trained TBAs disrupted their farming activities as they had less time to work on their farms. Most trained TBAs and pregnant women who were interviewed mentioned that incentives should be increased because in their new roles TBAs would have no other source of income. However, in Somaliland five years after the training, the incentives were withdrawn but reports confirm that some TBAs continued with their new roles and continue to refer and accompany women to health facilities for delivery.

So how should trained TBAs’ contributions be appropriately remunerated and sustained? Within both contexts MOH staff discussed strategies and policy initiatives to incorporate the new TBA role into the health system as Community Health Workers (Sierra Leone) or Lady Health Workers (Somaliland). This is a very positive development – formally integrating and supporting trained new-role TBAs within the health system is likely to promote positive relationships and outcomes. Consultation and fair and transparent approaches to remuneration will be critical in making this a success.

In summary, our findings from multiple perspectives across two different FCAS highlight the possible gains of new roles for TBAs through linking them with and integrating them into the formal health system. Ministry of Health staff, health workers, trained TBAs and communities all provided positive perspectives on the new role of TBAs. These findings may open further conversations and policy dialogue on how the formal health sector can leverage the non-clinical skills of TBAs to increase skilled birth attendance rates for marginalized rural women. Conversations on the integration of TBAs (in their traditional role) into the formal health sector has largely met with resistance by formal health sector actors [37]. Proponents of integration argue that TBAs are close to the community and provide vital links to reaching the community as demonstrated in our study while opponents fear the possibility of TBAs taking on more than they can handle, delaying referrals and causing further complications for women [35, 38–41]. Our qualitative analysis of different perspectives has highlighted that TBAs when engaged in well-defined and suitable largely non-clinical roles have major contributions to make to maternal and newborn health as close to community providers. Their potential is arguably critically important in promoting UHC in FCAS where human resources for health are additionally constrained and MCH needs particularly acute.

### Limitations

This is a purely qualitative study which captures and analyzes stakeholder’s perspectives on the new roles of trained TBAs and their perceived impact rather than actual health centre data confirming impact on maternal and child health outcomes and further health outcome data is required to underpin decisions around scale up. The confirmation of themes across two different contexts and the triangulation of perspectives (whereby views from women, TBAs and health staff largely confirmed each other) means that the results are likely to have a wider resonance and applicability. It was unfortunately beyond the scope and resources available for this study to conduct comparative qualitative research in areas where the new approach to TBAs was not being undertaken. It is possible that participant interactions were shaped by social desirability bias or the Hawthorne effect. Researchers in both cases, though independent worked in collaboration with the implementing partner and government. Participants may have felt they should report positive effects of the programme; we aimed to address this through developing trusting relationships with participants and appropriate in-depth probing. In addition, it is clear that integration of TBAs will require proper definition of roles, training and re-orientation, an acceptable placement and remuneration package and a monitoring and evaluation system to access effectiveness. It was beyond the scope of this study to address all of these issues which will require further exploration.

## Conclusion

Strategies to increase skilled birth attendance are particularly critical in fragile contexts where maternal and newborn health needs are great and human resources constrained. Close to community providers, such as TBAs, have strategic potential in reaching women in the community and linking them to skilled providers of maternity care. Training TBAs to refer and support women to deliver in health centres was positively received by a range of stakeholders in both Sierra Leone and Somaliland. Key challenges include sustainability and appropriate and fair remuneration. Evaluating similar approaches in different contexts will be important to generate further insights and perspectives into approaches to working with TBAs as close to community providers to improve maternal and newborn health. Even if our findings are not enough at this time to drive a policy shift towards renewed recognition and integration of TBAs as maternal health care providers in a role as health promoters, they add new insights and enrich the conversation around suitable and strategic roles for TBAs, which build on their embedded positionality.

## Additional files

Additional file 1: Sierra Leone IDI and FGD scripts Oct 2015. The scripts of the questions used by moderators during in-depth interviews and focus group discussions in Sierra Leone. (DOCX 49 kb)

Additional file 2: Somaliland IDI and FGD scripts Oct 2015. The scripts of the questions used by moderators during in-depth interviews and focus group discussions in Somaliland. (DOCX 39 kb)

Additional file 3: Topic guide for interviews with maternal health promoters. The questions asked during interviews with maternal health promoters following their training. (DOCX 21 kb)

Additional file 4: Training of Research Assistants in Sierra Leone. The description of the each of the 4 days of training received by research assistants in Sierra Leone. (DOCX 12 kb)

Additional file 5: Codes after merging in nVivo and Matrix- Sierra Leone. An example of the coding using during the analysis process. (DOCX 143 kb)

Additional file 6: Tree Map in nVivo - Sierra Leone. Mapping of the emerging themes. (DOCX 279 kb)

Additional file 7: Ethical Approvals LSTM. Letters of Ethical Approval from the Liverpool School of Tropical Medicine for the research in Sierra Leone and Somaliland. (DOCX 1375 kb)

## Abbreviations

ANC: Antenatal care
CHW: Community health worker
FCAS: Fragile and conflict affected states
FGD: Focus group discussion
FHCI: Free health care initiative
HPA: Health Poverty Action
HW: Health worker
IDI: In-depth interview
KI: Key informant interview
LSTM: Liverpool School of Tropical Medicine
MCH: Maternal and Child Health
MCHP: Maternal and Child Health Post
MDG: Millennium Development Goals
MHP: Maternal Health Promoter
MOH: Ministry of Health
NGO: Non-Governmental Organization
PHU: Peripheral Health Unit
RCT: Randomized controlled study
RHO: Regional Health Officer
SBA: Skilled Birth Attendant
SL: Sierra Leone
SMLD: Somaliland
TBA: Traditional Birth attendant
UHC: Universal Health Coverage
WHO: World Health Organization
