# Supplementary material for: Strengthening close to community provision of maternal health services in fragile settings: an exploration of the changing roles of TBAs in Sierra Leone and Somaliland
Source: BMC Health Serv Res. 2017 Jul 5;17:460. doi: 10.1186/s12913-017-2400-3 (PMC5498892; doi:10.1186/s12913-017-2400-3)
Supplement: Supplementary file 1 — Sierra Leone IDI and FGD scripts Oct 2015. The scripts of the questions used by moderators during in-depth interviews and focus group discussions in Sierra Leone. (DOCX 49 kb) [file 12913_2017_2400_MOESM1_ESM.docx]

**
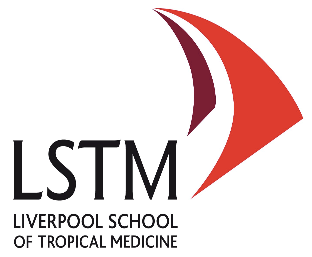
**

**IDI with maternal health promoters in Sierra Leone**

This study is been carried out to find out what you feel about the training you received, how it is going and the strengths and weaknesses. The participant information sheets that were distributed explained this in detail.

Your identity and information obtained will be treated with confidentiality.

Please can you tell me a little about yourself?

**Biodata:**

- Name
- Age
- How long you have been a traditional birth attendant?
- How far/near is your home from the health center?

1. Why did you decide to enroll for the training?
2. What did you think about the training?
3. Do you think the training has been useful so far and how?
4. Is the government supporting you in this role?
5. What problems have you experienced since you became an MHP?

- Relationship with health workers
- What women think of the new approach
- Referring women to health centers
- Problems with payment

1. What are the strengths of the training?
2. What do you think about women’s willingness to give birth in hospitals now compared to what it was before the training?
3. Would other TBAs you know be willing to undergo the same training? Why/why not?
4. Is there any other thing you may want to tell me about the training?

Thank you for participating in the study.

**
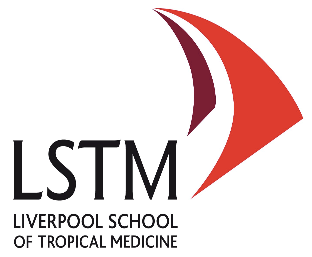
**

**IDI with Health Poverty Action staff in Sierra Leone**

Thank you for agreeing to take part in this study and your time and responses are greatly appreciated. This study is to find out your views and what you feel the opportunities and challenges of these maternal health promoters might be.

Your identity and information obtained will be kept confidentially.

**Overview data**:

Name

Position/ background in place of work

How long have you worked in this organization?

1. What do you know about the training of maternal health promoters and how involved is your organization in their training?

1. What do you think about the training of maternal health promoters?

- Reasons for training them
- Importance
- Problems
- Prospects

1. What contributions do you think the MHPs have made so far?
2. What do you think might be the opportunities and challenges faced by MHPs?

- Relationship with other health workers
- Problems with transportation
- How they feel about their new roles
- Remuneration

1. How have MHPs influenced the number of women delivering in health centers since their inception?
2. Are there any records of women supported or activities carried out by MHPs in the health facilities?
3. Has there been any evaluation done since inception of MHPs and what were the results?
4. Any other information you might want to add?

Thank you for participating in this study.

**
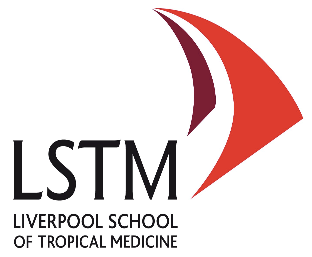
**

**IDIs with Health centre staff in Sierra Leone**

Thank you for agreeing to take part in this study and your time and responses are greatly appreciated. This study is to find out your views and what you feel the opportunities and challenges of these maternal health promoters might be.

Your identity and information obtained will be kept confidentially.

**Overview data**:

Name:

Position/ background in place of work:

How long have you worked in this health centre?

1. What do you know about the training of maternal health promoters?
2. How involved is your health centre in their training?
3. What do you think about the training of maternal health promoters?

- Reasons: Why do you think the training was conducted?
- Importance: what is the importance of this training?
- Problems: what problems do you for see with the MHPs?
- Prospects of the training

1. What contributions do you think the MHPs have made so far?
2. What do you think might be the opportunities and challenges faced by MHPs?

- Relationship with other health workers
- Problems with transportation
- How they feel about their new roles
- Remuneration

1. How have MHPs influenced the number of women delivering in health centers since their inception?
2. Are there any records of women supported or activities carried out by MHPs in the health facilities?

- How do you keep records of the women that are referred by the MHPs?
- Have the MHPs been consistent in sending women to the health centers?

1. Any other information you might want to add?

Thank you for participating in this study.

**
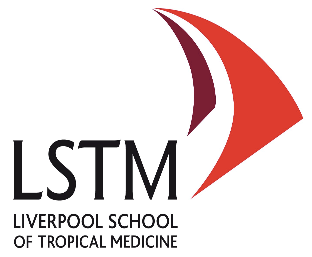
**

**Focus Group Discussions with pregnant/recently delivered women**

**WELCOME**: Welcome everyone. Thanks for agreeing to be part of the focus group. We appreciate your willingness to participate.

**INTRODUCTIONS:** Moderator, assistant moderator

**PURPOSE OF FOCUS GROUPS**: We are conducting this focus group to find out your views about the newly trained maternal health promoters. We need your input and want you to share your honest and open thoughts with us.

**GROUND RULES**

1. WE WANT YOU TO DO THE TALKING:

- We would like everyone to participate. I may call on you if I haven't heard from you in a while.

1. THERE ARE NO RIGHT OR WRONG ANSWERS:

- Every person's experiences and opinions are important.
- Speak up whether you agree or disagree.
- We want to hear a wide range of opinions.

1. WHAT IS SAID IN THIS ROOM STAYS HERE:

- We want folks to feel comfortable sharing when sensitive issues come up.

1. WE WILL BE TAPE RECORDING THE GROUP :

- We want to capture everything you have to say. We don't identify anyone by name in our report. You will remain anonymous.

1. Please tell us a little about yourselves, where you come from?
2. What was your experience like in your last pregnancy?
3. Did you get support in your pregnancy? If so, from who?
4. Have you or did you attend antenatal clinic in this pregnancy?

- Where did you attend antenatal?
- Why did you attend antenatal?
- How many times did you attend?

5. How do you feel about the new roles of the former traditional birth attendants?

6. Are you comfortable or not?

7. What support do MHPs have and how did they affect your pregnancy?

- Probe training?
- Probe impact?

1. How can MHPs be best supported in their role? Probe training, supervision
2. Is there any other thing you want to discuss or mention before we conclude?

Thank you for participating in this study.

**
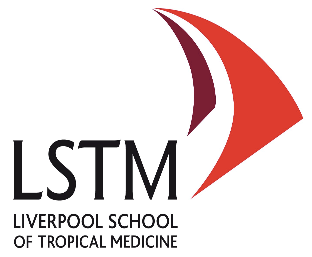
**

**IDI with Ministry of Health staff in Sierra Leone**

Thank you for agreeing to take part in this study and your time and responses are greatly appreciated. This study is to find out your views and what you feel the opportunities and challenges of these maternal health promoters might be.

Your identity and information obtained will be kept confidentially.

**Overview data**:

Name:

Position/background in place of work:

How long have you worked in this organization?

1. What do you know about the training of maternal health promoters

1. How involved is your organization in their training?

- How long were you involved in this training?

1. What do you think about the training of maternal health promoters?

- Reasons: Why do you think the training was conducted?
- Importance: what is the importance of this training?
- Problems: what problems do you for see with the MHPs?
- Prospects

1. What contributions do you think the MHPs have made so far?

- Any changes noticed in hospital deliveries since inception of MHPs?

1. How have MHPs influenced the number of women delivering in health centers since their inception?
2. Are there any records of women supported or activities carried out by MHPs in the health facilities?
3. Any other information you might want to add?

Thank you for participating in this study.
