# Supplementary material for: Strengthening close to community provision of maternal health services in fragile settings: an exploration of the changing roles of TBAs in Sierra Leone and Somaliland
Source: BMC Health Serv Res. 2017 Jul 5;17:460. doi: 10.1186/s12913-017-2400-3 (PMC5498892; doi:10.1186/s12913-017-2400-3)
Supplement: Supplementary file 2 — Somaliland IDI and FGD scripts Oct 2015. The scripts of the questions used by moderators during in-depth interviews and focus group discussions in Somaliland. (DOCX 39 kb) [file 12913_2017_2400_MOESM2_ESM.docx]

**SOMALILAND KEY INFORMANT INTERVIEW GUIDE**

*Instructions for the interviewer:*

1. Commence by reading out the participants’ information sheet for Key Informant Interview (translated into Somali) to the understanding of all.
2. Each participant must give his/her consent by signing the consent form before commencement of interviews.
3. Date of interview: _______________________________
4. Time started: _______________________________
5. Time ended: _______________________________
6. Place of interview: ­­­­­­­­­­­­­­­­­­­­­­­­­_______________________________
7. Interviewee’s ID: _______________________________
8. Interviewer’s name and position: _______________________
9. Tell me about yourself and your organization: (name of organization and your position/ roles)
10. What are the problems women commonly experience during pregnancy and childbirth in your community?
11. How are women catered for during pregnancy and childbirth (normal/ complicated) in your community? Probe who sees them first, who next etc? Where are they seen first, then subsequently?
12. How do traditional birth attendants assist pregnant women in your community during pregnancy, labour and after delivery? How do the TBAs make the initial contact with the women? Do the TBAs visit them at home? What exactly do they do for them?
13. Do you think this has changed recently? If yes, please explain to us how it has changed. Probe further; what aspects have changed? Ask for specific details. Do they escort patients with labour or complication to health facilities? Do they help mothers prepare for childbirth? What type of assistance do they provide? Do they visit homes and educate mothers on personal hygiene? Probe to get specifics about what they do now, how they do it and what is new.
14. Were you involved in the project ‘Improving the reproductive and sexual health of internally displaced people in “Marood Jeex”? If yes, could you tell us about your level of involvement and the role you played?
15. If you answered yes to 12 above, could you tell us about some specific activities within the project that you were involved in, for instance, mobilizing TBAs, training TBAs, assisting TBAs with specific tasks?
16. Please tell us more about some of the TBAs’ practices that impressed you. (Probe deeply for specifics. Respondents need to give examples with dates and places if possible)
17. Please tell us about some of the TBAs’ practices that you think they need to improve. (Probe deeply for specifics. Respondents need to give examples with dates and places if possible)
18. Reflecting on the project, which component do you think affected the TBAs’ present performance the most significantly? Explain why. (Probe deeply for specifics. Respondents need to give examples possibly with dates and places)
19. Which components of the project do you think needs improvement? Explain why and how. (Probe and allow respondent to reflect. Needs to talk about specific components of the TBA training/ incentivisation process bringing out the strengths and weaknesses.) Explore to gain insights into constraints and challenges faced by the project.
20. If you were given an opportunity would you want to work with this project again? Please explain what changes or modification you would like to make to the project.
21. Would you recommend that this project is carried out in another setting? If so, please give details.
22. Overall how will you rate the way the process involved in training, orienting and incentivising the TBAs during this project?
23. Overall, how will you rate the performance of the TBAs before and after their training?
24. Any further comments? (probe to get the final word of the interviewee both in regards to the process of training and incentivization and the TBA performances from his/her own perspective)

*Thank you for consenting to this interview.*

**SOMALILAND FOCUS GROUP DISCUSSION GUIDE FOR TBA**

FGD schedule

Facilitator’s comments: Facilitator: --------------------------

Note taker: --------------------------

Date: ----------------------------------

Location: -----------------------------

Time (start/finish): ----------------

*Instructions for the facilitator:*

1. Commence by reading out the participants’ information sheet for TBA (translated into Somali) to the understanding of all.
2. Each participant must give her consent by signing or thumb-printing the consent form before commencement of discussions.

| **Participant’s initials** | **Age** | **Years of work as TBA** | **No of children born (own)** | **Ethnicity** |
| --- | --- | --- | --- | --- |
| 1-  2-  3-  4-  5-  6-  7-  8- |  |  |  |  |
| **Distance to nearest health facility/maternity (km) or time to reach by quickest mean** | | | **Distance to nearest health facility with surgical capacity (km) or time to reach by quickest mean** | |
| 1-  2-  3-  4-  5-  6-  7-  8- | | |  | |
| **Training (trained or untrained)** | | | **Training provided by:** | |
| 1-  2-  3-  4-  5-  6-  7-  8- | | |  | |

1. **Birth Preparedness**

| **Notes for the facilitator** |
| --- |
| Key ideas to explore:   - How do TBAs view birth preparedness (preparations that women and their families make for the childbirth)? - How are TBAs involved in birth-preparedness? |

**Questions to ask:**

1. What preparations do women and their families make for the childbirth? Who is involved? When do they make these arrangements?
2. What do you do to help women prepare for childbirth? What do you discuss with women and/or their families during pregnancy? Do you advise women and their families to save some money to cover the cost of delivery? If so, do they comply with your advice?
3. Do women come to you during pregnancy for ante-natal check-ups? What kind of advice do you give the women? [Discussion should bring out issues like good nutrition/ feeding habits, personal and environmental hygiene, use of LLIN, taking their routine ANC medications, reporting worrying symptoms/ complications, avoiding harmful medications and traditional practices, etc)
4. **Care during Delivery**

| **Notes for the facilitator** |
| --- |
| Key ideas to explore:   - What do TBAs think of their roles during delivery (before and after their training)? - What do TBAs think of assisting deliveries in a health facility? |

**Questions to ask:**

1. What do you think of your role during delivery (before and after your training)?
2. Before your training, describe how you used to care for women during pregnancy, childbirth and after childbirth. Give examples.
3. After the training, describe how you care for women during pregnancy, childbirth and after childbirth. Give examples.
4. Tell us what you like most about your former role. What do you dislike about it?
5. What are the risks associated with attending deliveries in your former role?
6. Tell us what you like most about your new role. What do you dislike about it?
7. Where do women in your community prefer to deliver and why? Do you still deliver women at home? If so, what are their reasons for still preferring home births?
8. What do you think of assisting deliveries in a health facility? How do you get the women to a referral health facility? How do the women perceive you accompanying them? Give examples.
9. How do the staff of the health facility perceive you? Do they welcome you warmly when your escort or refer a patient? Do they provide feedback to you? Give example.
10. **Care for Obstetric Emergencies**

| **Notes for the facilitator** |
| --- |
| Key ideas to explore:   - Do TBAs still attend to women with obstetric complications? - What do TBAs know about obstetric complications during pregnancy and delivery? - How do they handle? What are their common practices (before and after the project)? |

**Questions to ask:**

1. What do you know about obstetric complications during pregnancy and delivery? (Focus on common complications like excessive bleeding during pregnancy, excessive bleeding after delivery, eclampsia/ convulsions, severe headaches, etc)
2. Among those complications, which ones are the most serious? (To mention with little or no prompting)
3. How do you care for the complication/s you see more often? What are your common practices (**before** and **after** the project)?
4. Do you refer or escort the women with complications to the health facilities; when and how? Describe a recent experience.
5. If yes, to which facilities do you make referrals and why? Do you accompany the women and/or make necessary arrangements with their families? Have you encountered any situation when you asked a woman to go to a facility but she did not manage to reach the facility in time? Why? How did you resolve it?
6. Do you know what happens to the women with complications after referral in a health facility? When do the women receive care? How? (including the behaviour of the staff) Discuss what normally happens when you accompany a patient to a health facility.
7. For those of you who accompanied the women to the health facility, how were you treated by the staff on duty? Were you warmly welcomed? Did they say anything to you? What did they say?
8. **Early Post-partum Care**

| **Notes for the facilitator** |
| --- |
| Key ideas to explore:   - What are current TBA practices during the early post-partum period? - How do TBAs perceive and practice their roles in early post-partum period? |

**Questions to ask:**

1. Do you follow up with women whom you have assisted during delivery (either attended or referred)? If yes, tell us how you do this. Please give instances when you did this.
2. How long after delivery do you see them and why? How often? What do you do during the visits? For the woman and/or for the newborn? Why?
3. If a woman has no problem during delivery and the baby is well, do you think they should go to a health facility? If so, why? If not, should they be checked up by someone else?
4. **Communication, Perception**

| **Notes for the facilitator** |
| --- |
| Key ideas to explore:   - How does communication take place among TBAs, women and health staff? - How do TBAs perceive their work? |

**Questions to ask:**

1. If the women need your assistance, how do they communicate with you?
2. How do you communicate with the health facility if they need further treatment/referral?
3. What do you feel about the value of your job **before** and **after** the training? Is your prestige lower or higher? How does that affect the way you do your job?
4. **Payment/incentives**

| **Notes for the facilitator** |
| --- |
| Key ideas to explore:   - How do TBAs perceive the incentives they receive? |

**Questions to ask:**

Generally, how are TBAs paid for the assistance they render to women during pregnancy and childbirth? Please comment.

*Thank you for taking the time to talk to us. Your answers have been very helpful. Is there anything you would like to discuss?*

**SOMALILAND FOCUS GROUP DISCUSSION GUIDE FOR COMMUNITY WOMEN**

FGD schedule

Facilitator’s comments: Facilitator: --------------------------

Note taker: --------------------------

Date: ----------------------------------

Location: -----------------------------

Time (start/finish): ----------------

*Instructions for the facilitator:*

1. Commence by reading out the participants’ information sheet for community women (translated into Somali) to the understanding of all.
2. Each participant must give her consent by signing or thumb-printing the consent form before commencement of discussions.

| **Participant’s initials** | **Age** | **Ethnicity** | **Literate (Yes/No)** | **No of children born (delivered by TBA/total)** |
| --- | --- | --- | --- | --- |
| 1-  2-  3-  4-  5-  6-  7-  8- |  |  |  |  |
| **Distance to nearest health facility/maternity (km) or time to reach by quickest route available** | | | **Distance to nearest health facility with surgical capacity (km) or time to reach by quickest mean** | |
| 1-  2-  3-  4-  5-  6-  7-  8- | | | 1-  2-  3-  4-  5-  6-  7-  8- | |

1. **Pregnancy & Birth Preparedness**

| **Notes for the facilitator** |
| --- |
| Key ideas to explore:   - How are women cared for during pregnancy? - What types of routine preparations are made for childbirth? - Who helps in planning and making preparations for birth? - Who is the decision-maker for delivery location and preparations for birth? - What is the role of TBAs in pregnancy care and birth preparedness? |

**Questions to ask:**

1. When you are pregnant do you receive care from TBAs? When? What? How?
2. When you are pregnant, do you and your family discuss where you’re going to deliver the baby?

*If yes:* Who’s involved in the conversation? Who initiates it? When, during the pregnancy do you start to discuss this issue? What is said? What are all the places for delivery that women and their families consider?

*If no:* Why not? Should you have this kind of discussion? How could a woman initiate a conversation like this with her family?

1. What preparations do you and your families make for childbirth? For yourself? For the new baby? Who is involved? What do you do? What do men do? How are female elders involved? When are these preparations made?
2. If you and your family have different ideas about where you should deliver or which preparations should be made, who makes the final decision? How much influence do you have over where you’re going to deliver?
3. Do nearby TBAs get involved in this discussion? How? At what point? Who informs the TBAs? Encourage to share recent experience if any.
4. **Care during Delivery**

| **Notes for the facilitator** |
| --- |
| Key ideas to explore:   - Where do women in your community prefer to deliver? - What are barriers to delivering in health facilities? - How are these overcome? - What roles do TBAs play in overcoming these barriers? |

**Questions to ask:**

1. Where do you and women in your community prefer to deliver? Why do you think some women deliver at home? What do they like about delivering at home? What do family members do to help?
2. What do TBAs do to help during delivery? What are the best parts about getting help from a TBA? Do TBAs refer you during delivery? If yes, where to and why? Are some women getting assistance from TBAs during deliveries – at TBA’s homes or at women’s homes? If yes, why do you think they prefer to deliver with TBAs and others deliver by themselves or with family members?
3. What do staff at health facilities do to help you during delivery? How do they help you to feel more comfortable? How do they treat the people who come with a woman to the health facility, like her family members or a TBA? Why do you think some women deliver at health facilities and others deliver their babies at home and/or with TBAs?
4. In your community, what do the husbands do when their wives go into labour? Does the husband provide any support like getting ready for referral if needed? In most cases, who makes the decision for referral?
5. At what point do you involve TBAs during delivery? When labour starts? When delivery delays? When complication develops? Who calls the TBA?
6. **Care for Obstetric Emergencies**

| **Notes for the facilitator** |
| --- |
| Key ideas to explore:   - What do women know about obstetric complications? - How do women feel about planning for obstetric emergencies? - What roles do TBAs play? |

**Questions to ask:**

1. In your community, what types of problems do women commonly have; during late pregnancy? During delivery? During the first few weeks after childbirth?
2. Among those problems, which do you think are the most serious? *For each problem ask:*
   1. Have any of you ever had that problem?
   2. Do you know anyone who had that problem?
   3. Do you know what causes that problem?
   4. What can happen to the woman who has that problem?
3. If a woman in your community is experiencing one of these problems, what can she and her family do? *Ask each of the following questions:*
   1. Is there anything that they can try at home to solve the problem? What? Why?
   2. Where can they go to get help in your community?
   3. Which health facilities can they go to?
   4. What kinds of arrangements do they make if they decide to go to a health facility?
   5. What kind of transportation can they take to get to a health facility?
   6. What do you think can happen once they get to a health facility?
4. Do women and their families ever make plans in advance in case problems like these occur?

*If yes:* What do they do? Why? Who is involved in discussion and making these arrangements? Are there other plans that you think they should make in advance? What would make it easier for women and their families to make these kinds of plans?

*If no:* Why not? Do you think that they should have this kind of discussion? How easy or difficult would it be to make these kinds of plans?

1. At what point do you involve/ inform TBAs? Who informs the TBA? How long does it take to reach the woman? What do they do on arrival?
2. **Early Post-partum Care**

| **Notes for the facilitator** |
| --- |
| Key ideas to explore:   - What are the current practices during the early post-partum period? - How do women view early post-partum period on a routine basis? - What roles do TBAs play? |

**Questions to ask:**

1. Does anyone visit your home in the first weeks after you give birth? Who? How soon after the delivery? What do they do? Are there any particular people who will not be allowed to visit your home so soon after you have given birth?
2. How soon after you give birth can you first leave your house? Can you leave the house whenever you want after childbirth? Why? Are there exceptions?
3. Do you think that you should go to a health facility after delivery to get a check-up for yourself and/or for baby? Why or why not? If you have a normal delivery and both mother and baby are well, should you go for a check-up for yourself? Why or why not? How soon after the birth should you go? Who should go with you? If you don’t go to a health facility, where do you go? Why?
4. **Communication**

| **Notes for the facilitator** |
| --- |
| Key ideas to explore:   - How do pregnant women receive information regarding health? - What communications take place between women and health staff, women and TBAs? |

**Questions to ask:**

1. How do you get health-related information? From family members? From friends? From elderly women in their families and society? From radio, newspaper, health talks, others?
2. If you listen to the radio, what are your favourite programmes? Why do you like them? At what times of the day do you listen to the program? Name the programmes.
3. If you need assistance, how do you communicate with health facility?
4. If you need assistance, how do you communicate with TBAs?
5. **Incentives**

| **Notes for the facilitator** |
| --- |
| Key ideas to explore:   - Do women pay TBAs for their services? |

1. How do women pay for the services of a TBA?
2. What happens if women are unable to pay TBAs?
3. Has anybody experienced that before? Give example.
4. **Perceptions and preferences**

| **Notes for the facilitator** |
| --- |
| Key ideas to explore:   - How do women compare TBAs in their new roles versus the old? - What are their preferences? |

Questions to ask

1. Do your prefer TBAs to assist with delivery at home or refer and escort you to a health facility?
2. Which one do you prefer? Why is it your preference?
3. In your next pregnancy, where will you deliver? And why?
4. Is there anything else you would like to discuss?

*Thank you for taking the time to talk to us. Your answers have been very helpful. Is there anything you would like to discuss?*
