# Supplementary material for: Strengthening close to community provision of maternal health services in fragile settings: an exploration of the changing roles of TBAs in Sierra Leone and Somaliland
Source: BMC Health Serv Res. 2017 Jul 5;17:460. doi: 10.1186/s12913-017-2400-3 (PMC5498892; doi:10.1186/s12913-017-2400-3)
Supplement: Supplementary file 3 — Topic guide for interviews with maternal health promoters. The questions asked during interviews with maternal health promoters following their training. (DOCX 21 kb) [file 12913_2017_2400_MOESM3_ESM.docx]

**Topic guide for interviews with maternal health promoters.**

This study is being carried out to find out what you feel about the training you received, how it is going and the strengths and weaknesses. Your identity and information obtained will be treated with confidentiality.

Please can you tell me a little about yourself?

Part 1: Overview data:

1. Name
2. Age
3. How long you have been a traditional birth attendant?
4. How far/near is your home from the health center?

Part 2

1. Please explain about your role as an MHP? Probe role, responsibilities, opportunities, challenges

1. What support is provided for MHPs? Probe training
2. Who organizes the training? How did you come to know about it? What did you think about being invited for training?
3. Why did you decide to enroll for the training?
4. What did you think about the training?
5. Do you think the training has been useful so far and how?
6. What problems have you experienced since you became an MHP?

- Relationship with health workers
- What women think of the new approach
- Referring women to health centers
- Problems with payment

8. What are the strengths of the training?

9. Would other TBAs you know be willing to undergo the same training? Why/why not?

10. Is there any other thing you may want to tell me about the training?

***Thank you for participating in the study.***

**Topic guide for Health Poverty Action staff**

Thank you for agreeing to take part in this study and your time and responses are greatly appreciated. This study is to find out your views and what you feel the opportunities and challenges of these maternal health promoters might be. Your identity and information obtained will be kept confidentially.

Overview data:

Name

Position/Background in place of work

How long have you worked in this organization?

What is your role in this organization?

What are the key priorities of Maternal and Child Health In Sierra Leone?

What is the role of the MHPs?

What are the strengths and weaknesses of the MHPs?

1. What do you know about the training of maternal health promoters and how involved is your organization in their training?

2. What do you think about the training of maternal health promoters?

- Reasons for training them
- Importance
- Problems
- Prospects

1. What contributions do you think the MHPs have made so far?
2. What do you think might be the opportunities and challenges faced by MHPs?

• Relationship with other health workers

- Problems with transportation
- How they feel about their new roles
- Remuneration

5. How have MHPs influenced the number of women delivering in health centers since their inception?

6. Are there any records of women supported or activities carried out by MHPs in the health facilities?

7. Has there been any evaluation done since inception of MHPs and what were the results?

8. Any other information you might want to add?

***Thank you for participating in the study.***

**Topic guide for Ministry of Health staff**

Thank you for agreeing to take part in this study and your time and responses are greatly appreciated. This study is to find out your views and what you feel the opportunities and challenges of these maternal health promoters might be. Your identity and information obtained will be kept confidentially.

Overview data:

Name

Position/Background in place of work

How long have you worked in this organization?

What is your role in this organization?

What are the key priorities of Maternal and Child Health in Sierra Leone?

What is the role of the MHPs?

What are the strengths and weaknesses of the MHPs?

What do you know about the training of maternal health promoters and how involved is your organization in their training?

How long were you involved in this training?

What do you think about the training of maternal health promoters?

Reasons: Why do you think the training was conducted?

Importance: what is the importance of this training?

Problems: what problems do you for see with the MHPs?

Prospects: What contributions do you think the MHPs have made so far?

Any changes noticed in hospital deliveries since inception of MHPs

How have MHPs influenced the number of women delivering in health centers since their inception?

Are there any records of women supported or activities carried out by MHPs in the health facilities?

Is there any other information you might want to add?

***Thank you for participating in the study.***

**Topic guide for focus group discussions with pregnant/recently delivered women**

**WELCOME:** Thanks for agreeing to be part of the focus group. We appreciate your willingness to Participate.

**INTRODUCTIONS:** Moderator, assistant moderator

**PURPOSE OF FOCUS GROUPS:** We are conducting this focus group to find out your views about the newly trained maternal health promoters. We need your input and want you to share your honest and open thoughts with us.

**GROUND RULES**

a. WE WANT YOU TO DO THE TALKING. We would like everyone to participate. I may call on you if I haven't heard from you in a while.

b. THERE ARE NO RIGHT OR WRONG ANSWERS Every person's experiences and opinions are important. Speak up whether you agree or disagree. We want to hear a wide range of opinions.

c. WHAT IS SAID IN THIS ROOM STAYS HERE We want folks to feel comfortable sharing when sensitive issues come up.

d. WE WILL BE TAPE RECORDING THE GROUP We want to capture everything you have to say. We don't identify anyone by name in our report. You will remain anonymous.

1. Please tell us a little about yourselves, where you come from?
2. What was your experience like in your last pregnancy?
3. Did you get support in your pregnancy? If so, from who?
4. Have you or did you attend antenatal clinic in this pregnancy? Where did you attend antenatal and why? How many times?

5. How do you feel about the new roles of the former traditional birth attendants?

6. Are you comfortable or not? What support do MHPs have and how did they affect your pregnancy? Probe training? Probe impact?

7. How can MHPs be best supported in their role? Probe training, supervision. Are there any other thing you want to discuss or mention before we conclude?

***Thank you for participating in the study.***

**Topic guide for health center staff**

Thank you for agreeing to take part in this study and your time and responses are greatly appreciated. This study is to find out your views and what you feel the opportunities and challenges of these maternal health promoters might be. Your identity and information obtained will be kept confidentially.

Overview data:

Name

Position/Background in place of work

How long have you worked in this organization?

What is your role in this organization?

What are the key priorities of Maternal and Child Health in Sierra Leone?

What is the role of the MHPs?

What are the strengths and weaknesses of the MHPs?

What do you know about the training of maternal health promoters and how involved is your organization in their training?

How long were you involved in this training?

What do you think about the training of maternal health promoters?

1. What do you know about the training of maternal health promoters and how involved is your health center in their training?

2. What do you think about the training of maternal health promoters?

- Reasons: Why do you think the training was conducted?
- Importance: what is the importance of this training?
- Problems: what problems do you for see with the MHPs?
- Prospects

1. What contributions do you think the MHPs have made so far?
2. What do you think might be the opportunities and challenges faced by MHPs?

- Relationship with other health workers
- Problems with transportation
- How they feel about their new roles
- Remuneration

5. How have MHPs influenced the number of women delivering in health centers since their inception?

6. Are there any records of women supported or activities carried out by MHPs in the health facilities?

- How do you keep records of the women that are referred by the MHPs?
- Have the MHPs been consistent in sending women to the health centers?

1. Is there any other information you might want to add?

***Thank you for participating in the study.***
