# Supplementary material for: Strengthening close to community provision of maternal health services in fragile settings: an exploration of the changing roles of TBAs in Sierra Leone and Somaliland
Source: BMC Health Serv Res. 2017 Jul 5;17:460. doi: 10.1186/s12913-017-2400-3 (PMC5498892; doi:10.1186/s12913-017-2400-3)
Supplement: Supplementary file 4 — Training of Research Assistants in Sierra Leone. The description of the each of the 4 days of training received by research assistants in Sierra Leone. (DOCX 12 kb) [file 12913_2017_2400_MOESM4_ESM.docx]

**Training of Research Assistants in Sierra Leone**

Training began as soon as we arrived at Kamakwie, which is in Sella Limba where HPA has an office in northern Bombali and also where I stayed through the study duration. The assistant program manager of HPA recruited three females as I specifically asked for females knowing majority of my participants were women. Brief interview was conducted and two women were selected. One spoke Susu and English and the other Limba and English. The training lasted for about four days and entailed the following:

Day 1. The scope of the study was explained to the research assistant/translator and payments agreed after which translator agreement forms were signed. The aim and objectives were then explained in detail to the translators as they had no prior knowledge about research. I had to explain about the new MHPs who are locally called “mammys”.

Day 2. I began teaching them how to ask open ended questions with examples such as Open-ended questions “ What do you do when you are sick?”, who takes care of you when you are pregnant?

Closed question- Do you go to the hospital when you are sick?

We (translator and researcher) practiced different questions together and decided to test the topic guides with the recorder.

Day 3. Translators were asked to write about themselves and translate into the various languages just to test their writing ability. We began translating the consent forms and participant information sheets, which we had to do together because I had to make sure information was the same as, contained in document.

Day 4. We continued the translation of the consent forms and participant information sheet the next day.
