# Supplementary figures and images for: Strengthening close to community provision of maternal health services in fragile settings: an exploration of the changing roles of TBAs in Sierra Leone and Somaliland
Source: BMC Health Serv Res. 2017 Jul 5;17:460. doi: 10.1186/s12913-017-2400-3 (PMC5498892; doi:10.1186/s12913-017-2400-3)

**CODES AFTER MERGING IN NVIVO AND MATRIX- SIERRA LEONE**


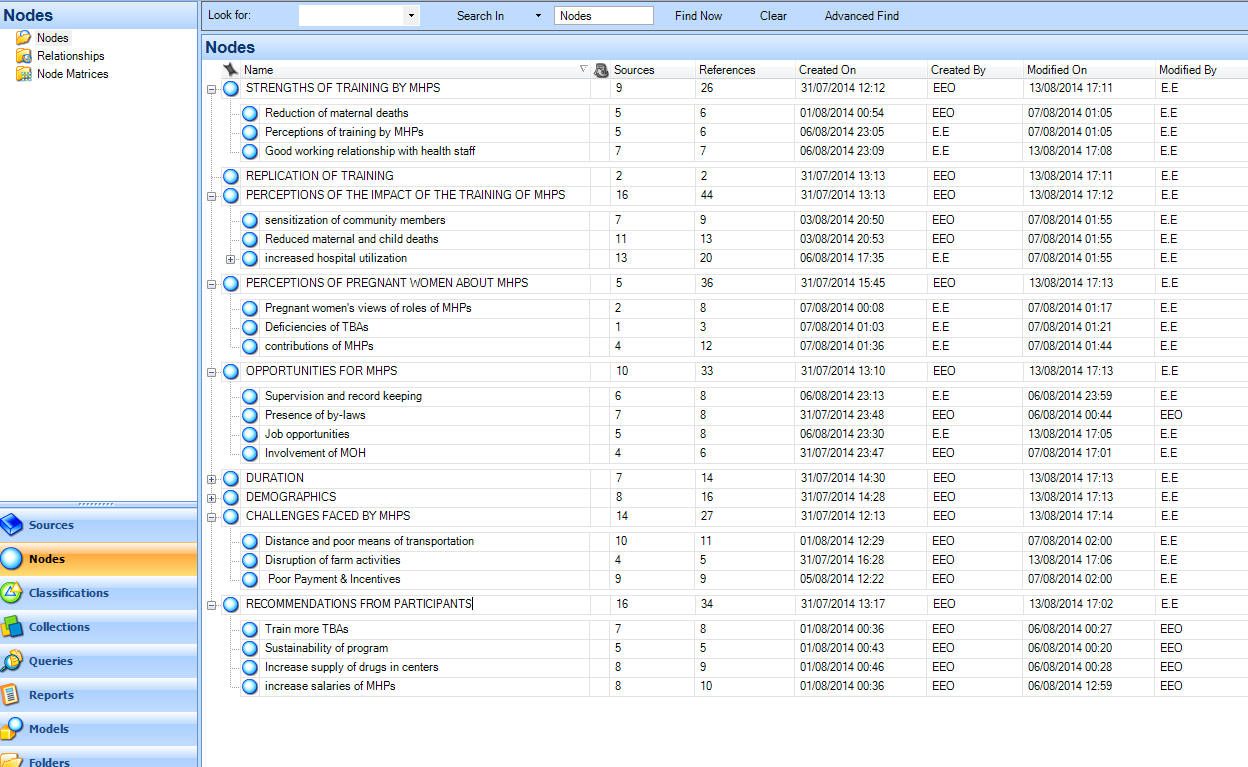

Supplement: Supplementary file 5 — Codes after merging in nVivo and Matrix- Sierra Leone. An example of the coding using during the analysis process. (DOCX 143 kb) [file 12913_2017_2400_MOESM5_ESM.docx]

**Supplementary File 6. Tree Map in nVivo - Sierra Leone**


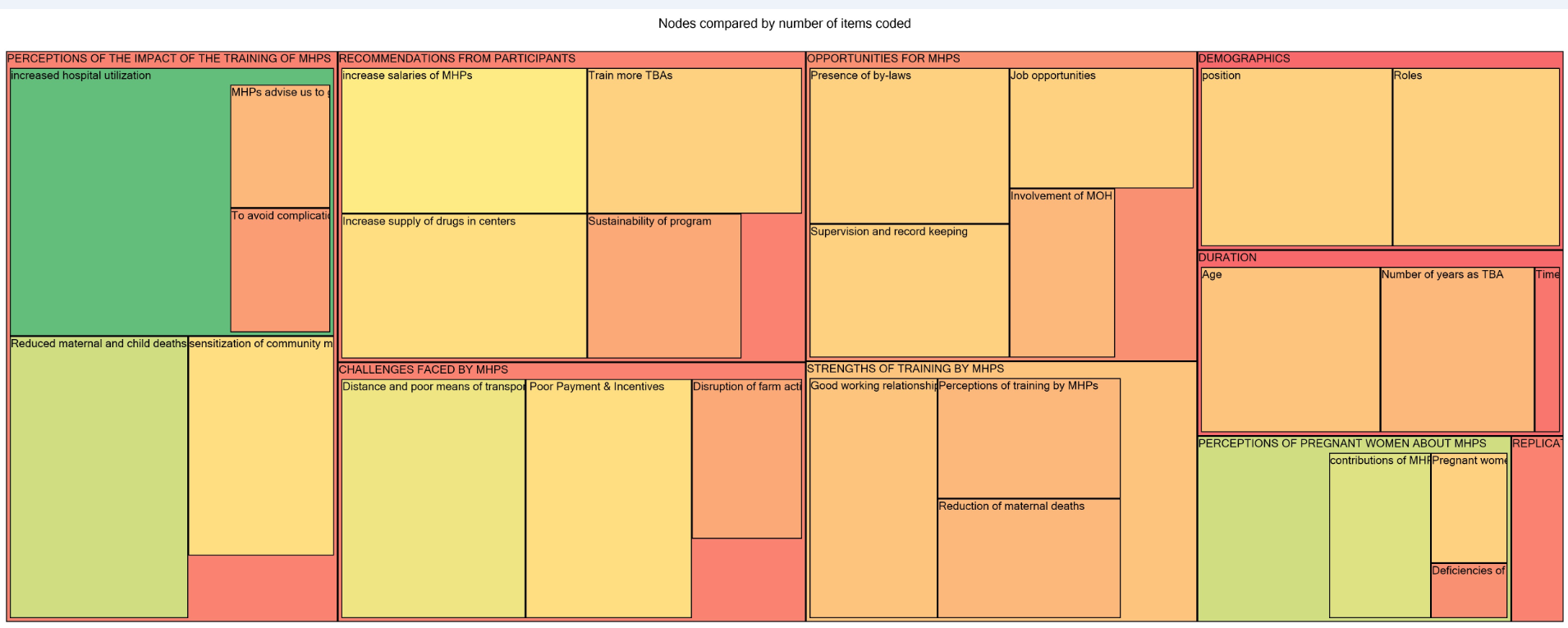

Supplement: Supplementary file 6 — Tree Map in nVivo - Sierra Leone. Mapping of the emerging themes. (DOCX 279 kb) [file 12913_2017_2400_MOESM6_ESM.docx]
