# Supplementary material for: Strengthening close to community provision of maternal health services in fragile settings: an exploration of the changing roles of TBAs in Sierra Leone and Somaliland
Source: BMC Health Serv Res. 2017 Jul 5;17:460. doi: 10.1186/s12913-017-2400-3 (PMC5498892; doi:10.1186/s12913-017-2400-3)
Supplement: Supplementary file 7 — Ethical Approvals LSTM. Letters of Ethical Approval from the Liverpool School of Tropical Medicine for the research in Sierra Leone and Somaliland. (DOCX 1375 kb) [file 12913_2017_2400_MOESM7_ESM.docx]

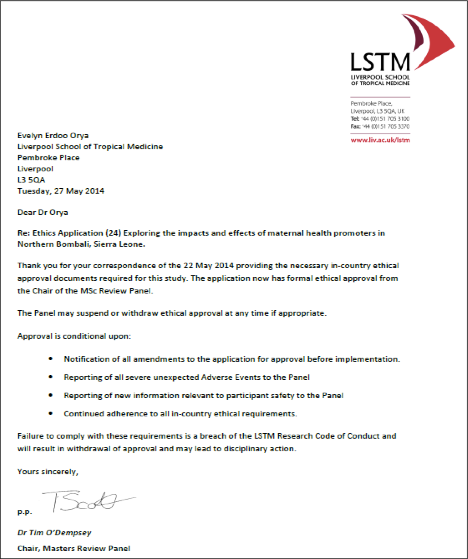


**LSTM ETHICAL APPROVAL – SIERRA LEONE**


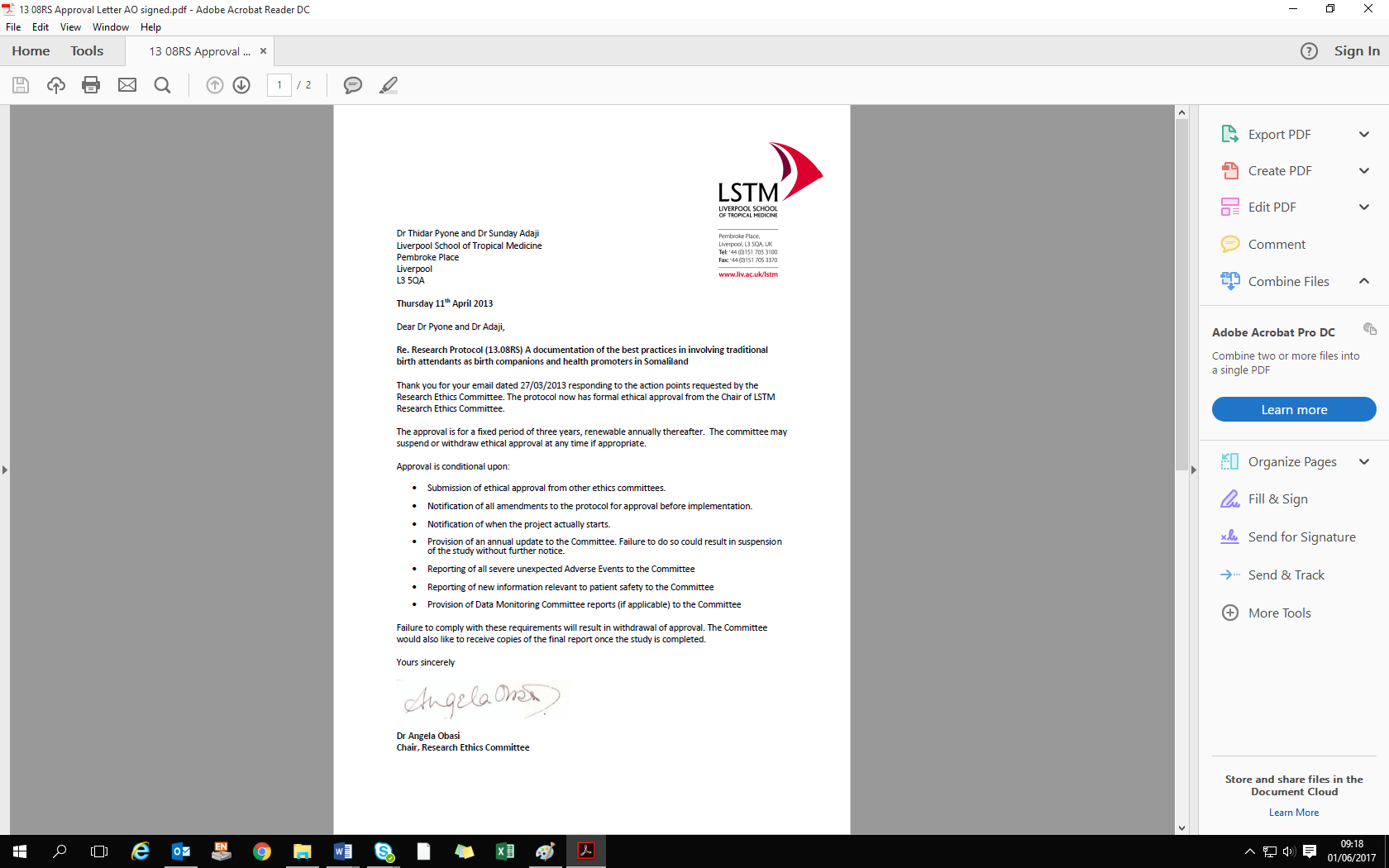


**LSTM ETHICAL APPROVAL – SOMALILAND**

**SIERRA LEONE ETHICAL APPROVAL**


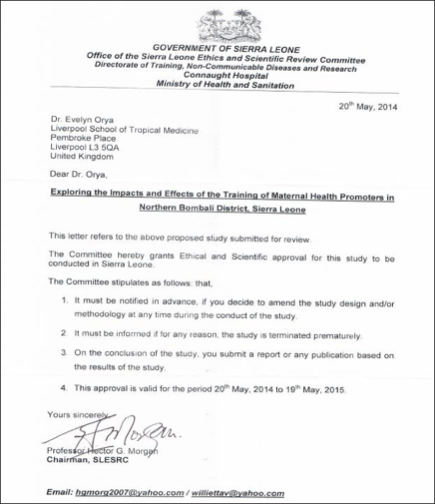


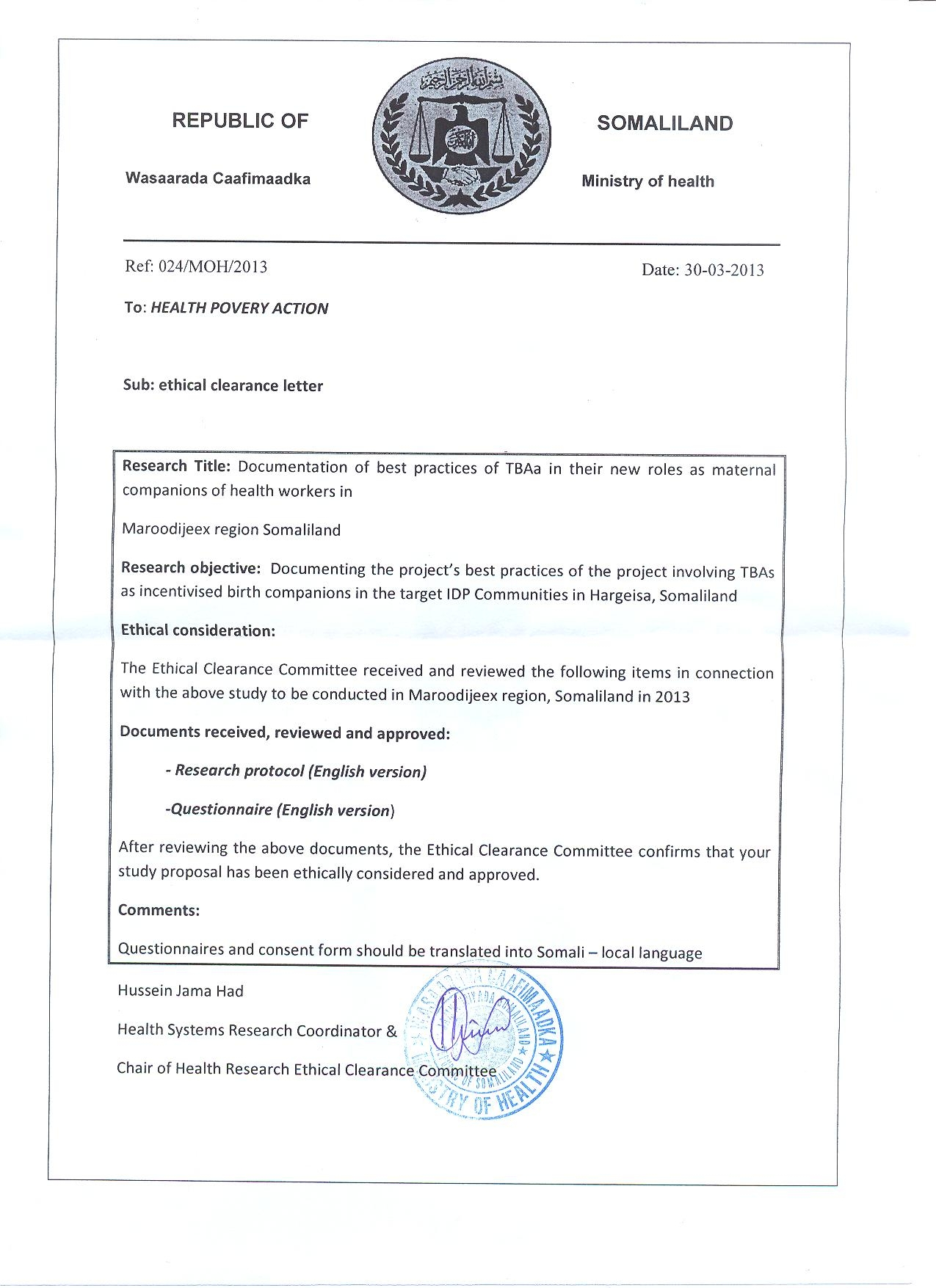


**SOMALILAND ETHICAL APPROVAL**
